# Sputum culture conversion and its predictors among drug-resistant pulmonary tuberculosis patients in eastern Ethiopia

**DOI:** 10.1093/inthealth/ihae059

**Published:** 2024-09-30

**Authors:** Mulugeta Gamachu, Alemayehu Deressa, Lemma Demissei Regassa, Miesso Bayu, Ibsa Mussa, Fekede Asefa Kumsa, Tariku Dingeta

**Affiliations:** School of Medicine, College of Health and Medical Sciences, Haramaya University, Harar, Ethiopia; Department of Public Health, Rift Valley University, Harar, Ethiopia; School of Public Health, College of Health and Medical Sciences, Haramaya University, Harar, Ethiopia; School of Public Health, College of Health and Medical Sciences, Haramaya University, Harar, Ethiopia; School of Medicine, College of Health and Medical Sciences, Haramaya University, Harar, Ethiopia; School of Public Health, College of Health and Medical Sciences, Haramaya University, Harar, Ethiopia; School of Public Health, College of Health and Medical Sciences, Haramaya University, Harar, Ethiopia; School of Population and Public Health, University of British Columbia, Vancouver, BC, Canada; School of Public Health, College of Health and Medical Sciences, Haramaya University, Harar, Ethiopia

**Keywords:** drug resistance, Ethiopia, predictors, sputum culture conversion, tuberculosis

## Abstract

**Background:**

Evidence of time to culture conversion is used to predict the time of cure from the disease and the overall drug-resistant tuberculosis (TB) treatment duration. Even though evidence about sputum culture conversion is enormous in TB treatment, no study has yet been done in our areas, where cases are common. The study aimed to assess the time to sputum conversion and its predictors among drug-resistant TB patients from October 2013 to September 2021 in eastern Ethiopia.

**Methodology:**

A retrospective cohort study was conducted in eastern Ethiopia among 273 drug-resistant TB patients who were treated from October 2013 to September 2021 at Dire Dawa City and Harari regional treatment centres. The Kaplan–Meier method was used to estimate the median time of sputum culture conversion. Cox proportional hazards regression was employed to detect the predictors of sputum culture conversion. An adjusted hazard ratio (aHR) with 95% confidence interval (CI) was used to determine the strength and significance of the association.

**Results:**

Of the 273 drug-resistant TB patients, the sputum culture of 216 (79.12%) patients became negative in a median time of 3 months (interquartile range 2–7). The time to sputum culture conversion was negatively associated with underweight (aHR 0.65 [95% CI 0.49 to 0.90]) and poor adherence (aHR 0.41 [95% CI 0.24 to 0.69]). The time to sputum culture conversion was also positively associated with patients resistant to two or more drugs (aHR 1.58 [95% CI 1.07 to 2.32]) and patients receiving a short treatment regimen (aHR 2.24 [95% CI 1.10 to 2.55]).

**Conclusions:**

A shorter culture conversion rate was observed compared with the median time recommended by the World Health Organization. Being underweight, poor adherence to treatment, resistance to two or more drugs and receiving a short treatment regimen were found to be predictors of time to sputum culture conversion. Implementing nutrition assessment, counselling and support of drug adherence may improve sputum culture conversion.

## Introduction

Globally, an estimated 10.6 million people developed TB in 2022, an increase from 10.3 million in 2021 and 10.0 million in 2020.^1^ The vast majority of TB cases, nearly 90%, are concentrated in 30 high-burden countries.^2,3^ Notably, approximately 3.3% of new TB cases and 17.7% of previously treated cases have been identified as multidrug-resistant (MDR) or rifampicin-resistant TB. Among the MDR-TB cases, 89.7% were reported in resource-poor countries.^3^ Ethiopia is classified among the 30 countries with a high burden of TB, TB/human immunodeficiency virus (HIV) and drug-resistant (DR)-TB from 2015 to 2020. Although the precise prevalence of DR-TB at the national level in Ethiopia remains uncertain, it is approximated that 2.7% of new TB cases and 14% of previously treated TB cases contribute to the burden.^4^ Furthermore, in 2019, MDR-TB was diagnosed in 0.71% of newly identified cases and in 12.0% of patients previously treated for TB.^5^

DR-TB continues to pose a global threat to public health, society and the economy. It remains a significant airborne infection that is resistant to many drugs. Approximately one-third of deaths were attributed to DR-TB caused by antimicrobial resistance, constituting 10% of all TB deaths.^2,6–8^ According to a global TB report, 600 000 cases of DR-TB were diagnosed resulting in 240 000 deaths in 2016, with most of these deaths occurring in Asia and Africa.^9^ In Ethiopia, DR-TB is a major public health issue that affects the productive segment of the population, with an estimated 5.8 new DR-TB cases per 1000 TB cases in 2016.^4,9^

In sub-Saharan Africa, the prevalence of DR-TB increased to 17% in 2021 from 12.6% in 2015 and it is commonly due to both physician error and poor patient compliance with treatment.^10,11^ Again the links between DR-TB, poverty, social protection, income per capita, indoor air pollution, undernutrition, diabetes, HIV, alcohol and smoking are well recognized in most countries, including Ethiopia.^4,5,12^

Time to sputum conversion helps to predict the duration of injectable drug use, the overall duration of DR-TB treatment and cure of the DR-TB.^12^ Additionally, predicting the initial conversion time helps for planning and implementing isolation.^5^ A delayed sputum conversion (i.e. a sputum conversion after 4 months of treatment initiation) is a precondition for suspecting extensively drug-resistant TB or treatment failure.^13^ Based on this, the World health Organization (WHO) highly recommends routine sputum conversion monitoring to assess treatment response and the use of 6-month sputum conversion status as a predictor of the final treatment outcome.^12^ The term ‘sputum culture conversion’ refers to the occurrence of two consecutive negative sputum cultures separated by a 30-day period. The date of the conversion is the first consecutive negative sputum culture.^12,13^ Previous studies have shown that the median time of sputum conversion is 2 months. Factors such as nutritional status, HIV status, diabetes, massive effusion of the lung on chest radiography, multiple drug resistance, history of previous treatment, type of treatment regimen and initial sputum smear grade are factors associated with a delay in time to sputum conversion.^12,14–18^

In 2017, the Ethiopian Federal Ministry of Health revised the National TB Control Program and introduced the guidelines on Programmatic Management of Drug-Resistant Tuberculosis, along with increasing medical and social services available for the management of DR-TB.^4^ In Ethiopia, TB control efforts have further stretched the growing health infrastructure, including the detection, diagnosis and management of drug resistance; monthly monitoring of sputum conversion status; and management of the comorbidities in DR-TB patients.^4^ Despite all these efforts, Ethiopia remains one of the high TB and DR-TB burden countries, where TB remains a substantial cause of morbidity and mortality.^4,19,20^

Although there have been some studies on sputum conversion in the country, the findings were inconsistent and missed essential variables such as types of regimens, laboratory findings and types of TB resistance, all of which may affect the time to sputum conversion.^4,19,20^ Even though evidence about sputum culture conversion is enormous in TB treatment, no study has yet been done in our area. In addition, TB is common in the current study area and the population has different characteristics. More information may also be required to guide policy aimed at enhancing the outcome of DR-TB treatment in Ethiopia. This study was aimed at estimating a median time to sputum culture conversion and its predictors among DR-TB patients in eastern Ethiopia.

## Methods

### Study design, area and period

A facility-based retrospective cohort study design was conducted in the Harari People's National Regional State and Dire Dawa City Administration, in east Ethiopia. Harari Region is located 526 km from Addis Ababa to the east. Based on the 2007 census conducted by the Central Statistical Agency of Ethiopia (CSA), Harari has a total population of 183 415 (92 316 men and 91 099 women). This region is the only one in Ethiopia where the majority of its population lives in an urban area (99 368 [54.18%]). The region has three public hospitals and eight health centres; among these, Amir Nur is the only health centre where DR-TB treatment is provided (since 2016).

Dire Dawa is located 515 km from Addis Ababa and 55 km from Harar. According to the CSA, the total population for 2019–2020 is estimated to be 506 639, and 68% of them are urban dwellers. The city has 2 public hospitals, 15 health centres and 36 health posts. The Dire Dawa Health Centre is only the health centre where DR-TB treatment is provided (since 2013).

### Study participants

Study participants were all DR-TB patients who were registered and started treatment in the study settings.

### Inclusion and exclusion criteria

All patients with complete treatment charts, culture positive for DR-TB at the baseline and who were treated for at least 2 months, from 2013 to 2021, were included in this study. DR-TB patients with incomplete charts, were baseline culture negative and who died or were lost follow-up <2 months after treatment initiation were excluded.

### Sample size determination

The sample size required for this study was determined with a sample size determination formula for survival analysis using Stata 14.2 (StataCorp, College Station, TX, USA) by considering three predictor variables from previously conducted studies in Oromia and Amhara Regions of Ethiopia.^15,21^ It was calculated using the following statistical assumptions: two-sided significant level (α) of 5%, power 80%, Zα/2 value at 95% CI 1.96, q1 (the proportion of subjects that are in group 1 [exposed]), q0 (the proportion of subjects that are in group 2 [unexposed]), 1−q1, hazard ratio (HR) and the probability of an event (E). Finally, the maximum selected sample size for this study was 237. The number of patients was small and close to the calculated sample size, so the study was conducted among all patients treated in the regions during the specified period, which was 273 (Table 1).

**Table 1.** Sample sizes for variables associated with sputum culture conversion

Variables	aHR	Probability of event	Event	Sample size
Abnormalities without cavitation	0.55	0.825	66	89
HIV negative	0.66	0.855	182	237
Treatment composition	0.57	0.855	100	130

### Data collection, laboratory sputum sample processing and quality control

Nine years (2013–2021) of data regarding DR-TB were extracted from medical charts of patients at Dire Dawa and Amir Nur DR-TB treatment health centres using a predefined checklist. The checklist was adapted from the results of similar studies^14,16,22,23^ and in line with the patients’ records. The basic sociodemographic characteristics, baseline and follow-up clinical characteristics and microbiological and laboratory results were extracted by reviewing TB registration books and cards of patients by trained health professionals.

Baseline and monthly follow-up microbiological data such as sputum smear microscopy, culture growth and drug susceptibility test results were also extracted from TB registration books, follow-up charts and electronic databases in the regions.

*Mycobacterium tuberculosis* (MTB) was isolated from sputum cultures using Löwenstein–Jensen medium and the results were documented as follows: no growth (none), actual number (1–9 colonies), 1+ (10–100 colonies), 2+ (>100–200 colonies), 3+ (>200 colonies), positive for other mycobacteria (other mycobacterial growth), contaminated (contaminated) and positive for MTB and contamination. All isolates of MTB were subjected to phenotypic drug-susceptibility testing (DST) using the concentration method and performed for first-line anti-TB drugs according to WHO guidelines.^24^ The Xpert MTB/RIF Assay was used for detection of MTB and rifampicin resistance directly from the sputum without the need for prior smear examination. Moreover, sputum smear grading was collected based on the number of acid-fast bacilli (AFB) observed and reported; negative indicated no AFB/100 high power field (HPF), scanty (1–9 AFB/100 HPF), 1+ (10–99 AFB/100 HPF), 2+ (1–9 AFB/HPF) and 3+ (>9 AFB/HPF). All patients included in our study had baseline drug susceptibility tests for first-line drugs using the Xpert MTB/RIF Assay and phenotypic DST or line probe assay.

A 2-day training was given for both data collectors and supervisors on the objective of the study and how to review the documents as per the data extraction checklist. All the checklists were reviewed daily by the supervisor. Double data entry was performed on 20% to check the consistency of the data and to correct mismatches. All microbiological sampling and testing were performed according to the national and WHO guidelines.

### Operational definitions and measurements

- Time to sputum culture conversion: the time to achieve two consecutive negative sputum cultures taken at least 30 days apart after an initial positive culture; the specimen collection date of the first negative culture is used as the date of conversion. The median sputum conversion time was measured on a monthly scale in this study.
- DR-TB: a type of TB that occurs when bacteria become resistant to the drugs used to treat TB diseases.
- Mono-resistant TB: TB resistant to one first-line anti-TB drug.
- MDR-TB: TB resistant to both isoniazid and rifampicin.
- DST: in vitro testing using either phenotypic methods to determine susceptibility or molecular techniques to detect resistance-conferring mutations to a medicine.
- New case: a newly registered episode of TB in a patient who has never been treated for TB or has taken anti-TB medicines for <1 month.
- Previously treated: patients who have received ≥1 month of anti-TB medicines. Previously treated cases may have been treated with a first-line regimen for drug-susceptible TB or a second-line regimen for DR-TB.
- Treatment outcome: defined as successful if the patient is cured or has completed treatment and unsuccessful if the patient died, failed treatment or was lost to follow-up (based on the 2008 WHO definition).
- Censored: when a culture result has not been converted for an individual during the study period; these may occur due to treatment discontinuation, death before conversion or when the patient is transferred to another facility.
- Adherence: defined as taking >90% of medications under conditions of direct observation by another person.^25^ In this study treatment adherence was measured by both subjective measurements obtained by asking patients, family members, caregivers and physicians about the patient's medication use and objective measurements obtained by counting pills and recording on the patient's follow-up chart as good, fair or poor.

### Data processing and analysis

Data were cleaned and checked for consistency and completeness. Data entry was done using EpiData version 3.1 (EpiData Association, Odense, Denmark) and exported to Stata 14.2 for further statistical analysis. Descriptive statistics such as percentages, medians, means, standard deviations (SDs) and interquartile ranges (IQRs) were computed. The median time to sputum culture conversion was estimated using the Kaplan–Meier test and the logrank test was used to compare survival time between groups of categorical variables. All variables with a p-value <0.25 in the bivariate analysis were included in the multivariable survival model analysis. In the multivariable analysis, Cox proportional hazards regression was used to detect the independent predictors of sputum culture conversion. The Kaplan–Meier curve and Schoenfeld residuals were used to check the proportional hazard assumptions. The adjusted hazard ratio (aHRs) with 95% confidence interval (CI) was used to identify the association between independent variables and sputum culture conversion. Statistical significance was declared at p<0.05 for the final analyses.

## Results

### Sociodemographic and behavioural characteristics

A total of 273 patients met the inclusion criteria and were included in the final analysis. The mean age of the study participants was 31 y (SD 13.7; range 6–76). Most participants were male (171 [62.24%]) and more than half (144 [52.75%]) of the study participants were from Dire Dawa. Less than half (128 [46.89%]) of patients were referred from general hospitals. The majority (236 [86.4%]) of patients were from urban residences. Ninety-two (33.70%) participants were single and 47 (17.22%) patients were students. Sixty-seven patients (24.54%) had a history of alcohol use, 89 (32.60%) had a history of cigarette smoking and 108 (39.56%) had a history of Khat chewing at baseline (Table 2).

**Table 2.** Sociodemographic and behavioural characteristics of DR-TB patients in Harari and Dire Dawa, eastern Ethiopia, from 2013 to 2021 (N=273)

Variables	Categories	n	%
Age (years)	≤14	16	5.86
	15–20	147	53.85
	31–45	64	23.44
	46–60	40	14.65
	>60	6	2.2
Sex	Male	171	62.64
	Female	102	37.36
Region of patients	Harari	32	11.72
	Dire Dawa	144	52.75
	Oromia	25	9.16
	Somali	68	24.91
	Other^a^	4	1.47
Residence	Urban	236	86.45
	Rural	37	13.55
Current marital status	Single	92	33.70
	Married	44	16.12
	Divorced	9	3.30
	Widowed	2	0.73
	Unknown	126	46.15
Education status	No formal education	37	13.55
	Primary (1–8)	74	27.11
	Secondary (9–12)	97	35.53
	≥College	65	23.81
Occupation	Government employee	20	7.33
	Self-employed	32	11.72
	Student	47	17.22
	Farmer	20	7.33
	Other^b^	16	5.6
	Unknown	138	50.4
Referring health facility	Comprehensive specialized hospital	23	8.42
	General hospital	128	46.89
	Primary hospital	24	8.79
	Health centre	98	35.90
Year of registration	2013–2015	98	35.89
	2016–2018	83	30.42
	2019–2021	92	33.69
History of alcohol use	No	206	75.46
	Yes	67	24.54
History of cigarette smoking	No	184	67.40
	Yes	89	32.60
History of khat chewing	No	165	60.44
	Yes	108	39.56

^a^Addis Ababa and Amhara.

^b^Drivers and street children.

### Clinical characteristics

Of the 273 patients with initial culture-positive results, 58 (21.25%) were new TB patients, 97 (34.43%) were on re-treatment after failure on the first-line anti-TB regimen, 58 (21.25%) were resistant to both isoniazid and rifampicin and 215 (78.75%) were resistant to only rifampicin. The majority of patients (204 [74.73%]) were treated with a standardized long DR-TB regimen. Fifty-one (18.68%) of the patients were HIV positive, 24 (10.81%) were living with comorbidities and the most common comorbidity was diabetes mellitus (DM) (14 [5.13%]). Nearly two-thirds (178 [65.20%]) had a body mass index (BMI) <18.5 kg/m^2^, 186 (68.13%) had good adherence to anti-TB treatment, 115 (42.12%) were cured and 30 (10.99%) died while they were being treated (Table 3).

**Table 3.** Frequencies and percentages of baseline clinical characteristics of DR-TB patients (n=273) in Harari and Dire Dawa, eastern Ethiopia, from 2013 to 2021

Variables	Categories	n	%
Registration group	New	58	21.25
	Relapse	71	26.06
	After the failure of the new FLD	37	13.55
	After the failure of treatment FLD	97	34.43
	Transfer in	13	4.76
Resistance type	Rifampicin-resistant TB	215	78.75
	MDR-TB	58	21.25
Regimen eligibility	Standardized long DR-TB treatment	204	74.73
	Individualized long DR-TB treatment	43	15.75
	Short DR-TB treatment	26	9.52
HIV status	Positive	51	18.68
	Negative	222	81.32
Comorbidity conditions other than HIV	Yes	24	10.81
	No	198	89.19
Baseline BMI categories	<18.5 kg/m^2^	178	65.20
	≥18.5 kg/m^2^	95	34.80
Baseline chest radiography	Unilateral lung cavity	14	5.13
	Bilateral chest cavity	11	4.03
	Abnormal without cavity	172	63.00
	Massive effusion	16	5.86
	Clear lung	37	13.55
	Not done	23	8.42
Baseline ECG result	Normal	156	57.14
	Abnormal	6	3.30
	Not done	108	39.56
Status of adherence to treatment	Good	186	68.13
	Fair	54	19.78
	Poor	33	12.09
Outcome of patients	Cured	115	42.12
	Completed	66	24.18
	Died	30	10.99
	LFU	24	8.79
	Not evaluated	38	13.92

FLD: first-line drug; LFU: lost to follow-up.

### Microbiological and laboratory test results

The majority (219 [80.22%]) of study participants had 1+ baseline sputum smear results and 178 (65.20%) had 1+ culture results at baseline. Similarly, 240 (87.91%) and 245 (94.25%) had a normal white blood cell count (4500–11 000/mm^3^) and red blood cell count (4.0–5.6 million/mm^3^) at the baseline, respectively. Thirteen (5%) patients had low (<3.5 mmol/l) potassium levels, 12 (4.39%) had low (<136 mmol/l) nitrogen levels and 17 (6.22%) participants had high baseline creatinine levels (Table 4).

**Table 4.** Baseline microbiological and laboratory test results of DR-TB patients (n=273) in Harari and Dire Dawa, eastern Ethiopia, from 2013 to 2021

Variables	Categories	n	%
Baseline smear result	Scanty	10	3.66
	1+	219	80.22
	2+	31	11.36
	3+	13	4.76
Baseline culture result (actual count of culture)	1–9	66	24.18
	10–99 (1+)	178	65.20
	>100 (2++)	29	10.62
Baseline WBC result	High (>11 000/mm^3^)	13	5.00
	Normal (4500–11 000/mm^3^)	245	94.23
	Low (<4500/mm^3^)	2	
Baseline RBC result	High (>5.6 million/mm^3^)	3	
	Normal (4.0–5.6 million/mm^3^)	240	93.38
	Low (<4.0 million/mm^3^)	14	5.45
Baseline K^+^ level	Normal (3.5–5 mmol/l)	116	82.36
	Low (<3.5 mmol/l)	13	9.23
	High (>5 mmol/l)	11	7.86
Baseline Na^+^ level	Normal (136–146 mmol/l)	76	79.17
	Low (<136 mmol/l)	12	12.5
	High (>146 mmol/l)	8	8.33
Baseline creatinine	High (>1.5 mg/dl)	17	7.08
	Normal (0.7–1.5 mg/dl)	220	91.67
	Low (<0.7 mg/dl)	3	

### Time to sputum culture conversion

**Figure 1.**
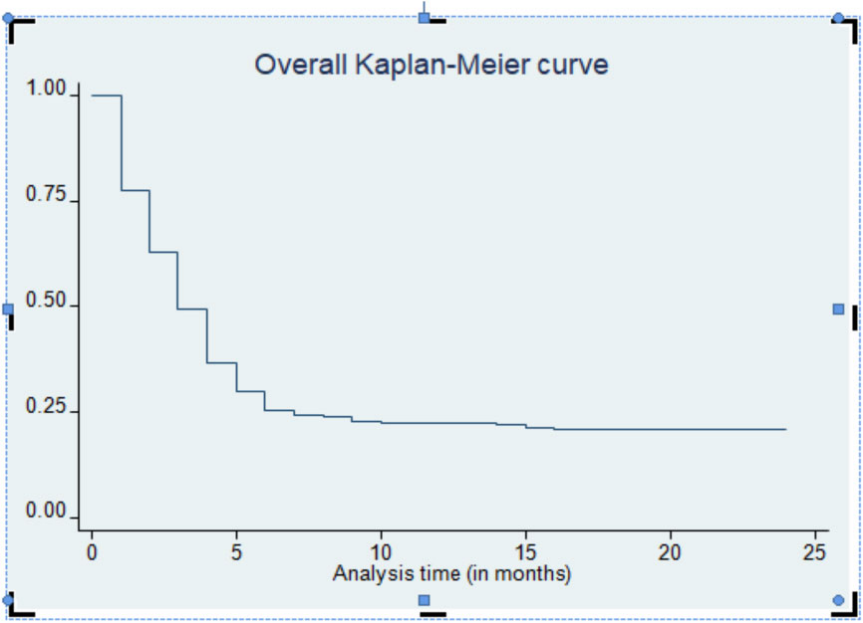
The median time of sputum culture conversion among DR-TB patients in Harari and Dire Dawa, eastern Ethiopia, from 2013 to 2021.

Of 273 study subjects who were culture positive at baseline, 216 (79.12%) became sputum culture negative for MTB within a median time of 3 months (IQR 2–7) (Figure 1).

**Figure 2.**
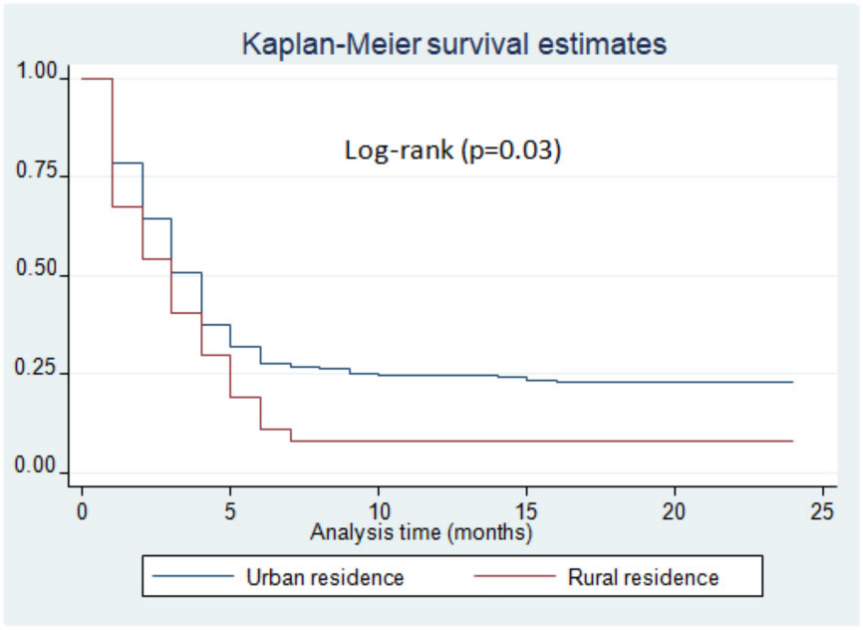
Sputum culture conversion time with patient's place of residence among DR-TB patients in Harari and Dire Dawa, eastern Ethiopia, from 2013 to 2021.

**Figure 3.**
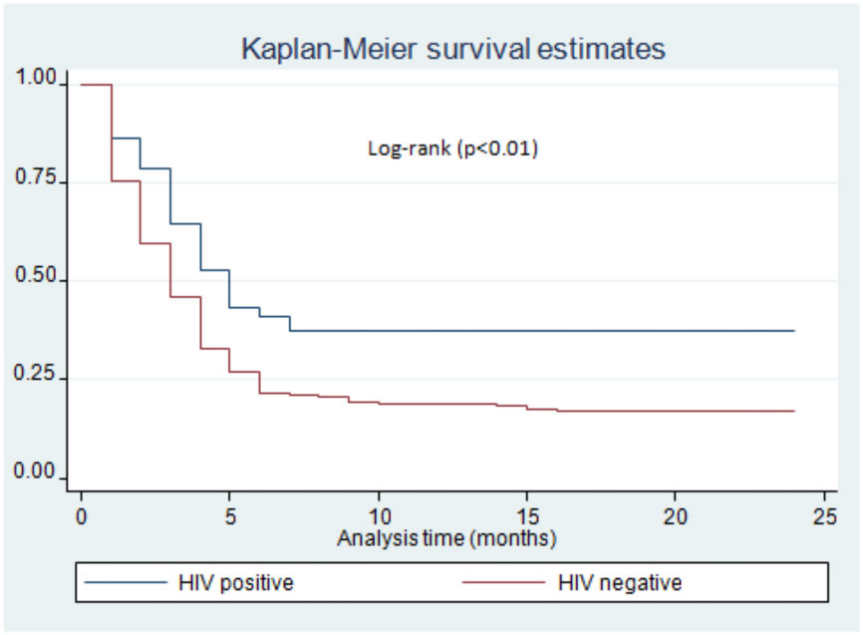
Sputum culture conversion time among DR-TB patients based on HIV/AIDS status in Harari and Dire Dawa, eastern Ethiopia, from 2013 to 2021.

**Figure 4.**
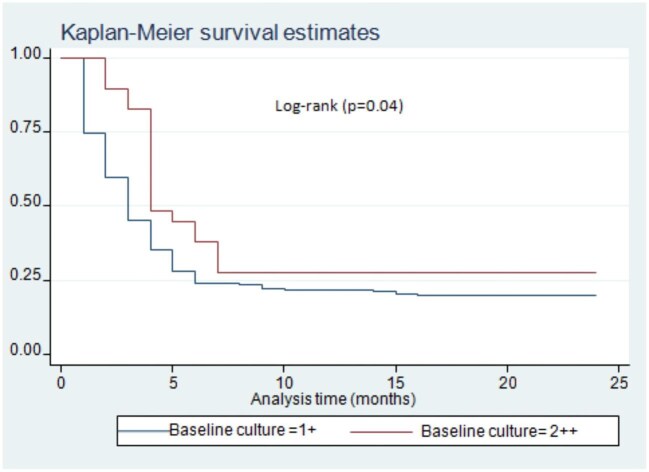
Time to sputum culture conversion based on baseline culture results among DR-TB patients in Harari and Dire Dawa, eastern Ethiopia, from 2013 to 2021.

**Figure 5.**
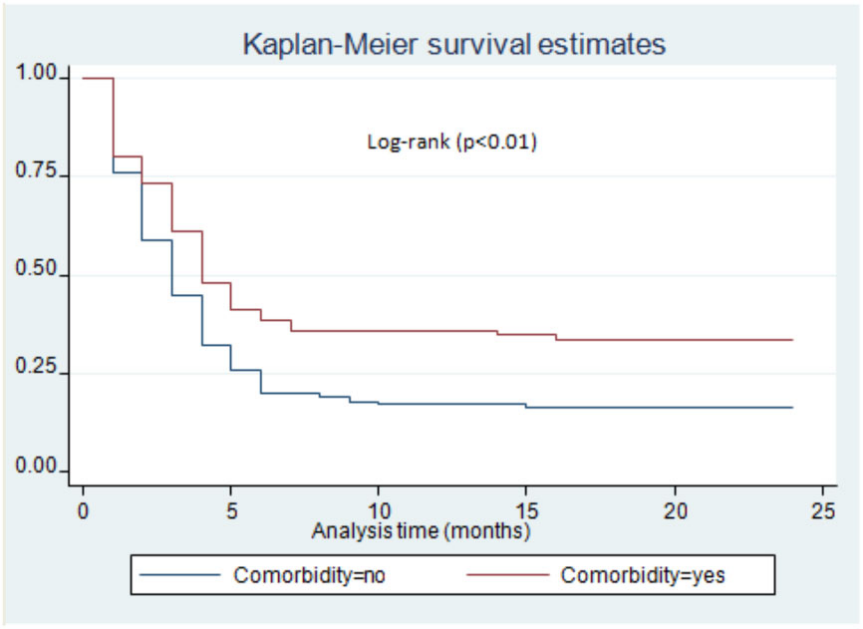
Time to sputum culture conversion with patient's HIV/AIDS status among DR-TB patients in Harari and Dire Dawa, eastern Ethiopia, from 2013 to 2021.

Patients who come from rural areas had a delayed rate of culture conversion on logrank test (p=0.03) (Figure 2). There was a difference in sputum culture conversion time among DR-TB patients with or without HIV/acquired immunodeficiency syndrome (AIDS) infection. Accordingly, DR-TB patients with HIV/AIDS co-infection had a delayed time to sputum culture conversion compared with HIV-negative patients (p<0.01) (Figure 3). Moreover, there was a significant difference in the time to sputum culture conversion based on the patients’ baseline culture results. The DR-TB patients who had a baseline culture >100 (2++) had a delayed sputum culture conversion time compared with patients who had a baseline culture ≤99 (1+) (p=0.04) (Figure 4). The DR-TB patients with a different comorbidity other than HIV/AIDS had a longer sputum culture conversion than patients without comorbidity on the logrank test (p<0.01) (Figure 5).

### Predictors of time to sputum culture conversion

Patients’ sociodemographic and behavioural characteristics, such as residence; education status; history of alcohol, cigarette or khat use; and clinical information such as type of resistance, regimen eligibility, baseline BMI, HIV/AIDS status, comorbidity conditions, baseline chest X-ray results, type of resistance, regimen eligibility, baseline culture result and status of drug adherence were assessed for a possible difference in the median time of sputum culture conversion using the logrank test. In the bivariable model, variables such as residence, education status, alcohol consumption history, history of smoking, history of khat chewing, type of resistance, treatment regimen, baseline BMI, HIV co-infection status, comorbidity other than HIV, baseline chest X-ray result, baseline culture result and status of drug adherence were significant at p<0.25 and included in the final multivariable model.

In the final multivariable Cox regression analysis, the study found that the type of resistance, regimen eligibility, baseline BMI categories and status of drug adherence were statistically significant predictors of sputum culture conversion. The hazard of sputum culture conversion among patients with baseline BMI <18.5 kg/m^2^ was lower by 35% compared with those with BMI ≥18.5 kg/m^2^ (aHR 0.65 [95% CI 0.49 to 0.90]). The hazard of sputum culture conversion among patients who had poor drug adherence was 59% lower than in patients who had good drug adherence (aHR 0.41 [95% CI 0.24 to 0.69]). In contrast, DR-TB patients who were resistant to two or more first-line TB drugs had a 58% greater chance of sputum culture conversion (aHR 1.58 [95% CI 1.07 to 2.32]) compared with patients resistant to rifampicin only. Furthermore, patients receiving short DR-TB regimens had a 24% higher probability of sputum culture conversion (aHR 2.24 [95% CI 1.10 to 2.55]) compared with patients receiving standardized long DR-TB regimens (Table 5).

**Table 5.** Bi variable and multivariable analysis of sputum culture conversion predictors among DR-TB patients in Harari and Dire Dawa, eastern Ethiopia, from 2013 to 2021

		Culture conversion			
Variables	Categories	Converted (event)	Not converted (censored)	Crude HR (95% CI)	aHR (95% CI)
Residence	Urban	182	54	1	1
	Rural	31	6	1.43 (0.99 to 2.07)	1.23 (0.83 to 1.84)
Education status	No formal education	26	11	1	1
	Primary (1–8)	59	15	1.61 (0.83 to 3.16)	1.71 (0.84 to 3.48)
	Secondary (9–12)	83	14	1.53 (0.79 to 2.90)	1.69 (0.83 to 3.43)
	≥College	56	9	1.28 (0.58,2.83)	1.56 (0.68,3.58)
History of alcohol use	No	167	39	1	1
	Yes	49	18	0.81 (0.58 to 1.1)	0.78 (0.56 to 1.37)
History of smoking	No	149	35	1	1
	Yes	67	22	0.83 (0.63 to 1.11)	1.27 (0.77 to 2.08)
History of khat chewing	No	133	32	1	1
	Yes	83	25	0.82 (0.62 to 1.08)	0.75 (0.49 to 1.17)
Type of resistance	Resistant to rifampicin only	165	50	1	1
	Resistant to two or more FLDs	51	7	1.65 (1.16 to 2.35)	1.58 (1.07 to 2.32)*
Treatment regimen	Standardized DR-TB regimen	161	43	1	1
	Individualized DR-TB regimen	35	8	0.93(0.66, 1.33)	1.05 (0.70,1.58)
	Short DR-TB regimen	20	6	1.74 (0.93 to 3.17)	2.24 (1.10 to 2.55)*
Baseline BMI	≥18.5 kg/m^2^	80	15	1	1
	<18.5 kg/m^2^	136	42	0.74 (0.56 to 0.97)	0.65 (0.49 to 0.90)**
HIV co-infection status	Positive	32	19	1	1
	Negative	184	36	1.68 (1.16 to 2.45)	1.03 (0.62 to 1.75)
Comorbidity other than HIV	No	166	32	1	1
	Yes	18	6	0.65 (0.47 to 0.88)	0.72 (0.47 to 1.10)
Baseline chest X-ray result	Unilateral lung cavity	25	3	1	1
	Bilateral lung cavity	8	5	0.56 (0.25 to 1.25)	0.70 (0.30 to 1.64)
	Abnormal without cavity	137	35	0.97 (0.63 to 1.49)	0.98 0.61 to 1.59)
	Clear lung	29	8	0.92 (0.53 to 1.56)	1.06 (0.59 to 1.92)
	Not done	17	6	0.73 (0.39 to 1.36)	0.63 (0.32 to 1.24)
Baseline culture result	Actual count (1–9)	47	19	1	1
	1+	148	30	1.36 (0.97 to 1.89)	1.32 (0.93 to 1.88)
	2++	21	8	0.83(0.49 to 1.39)	0.83 (0.47 to 1.43)
Drug adherence	Good	156	30	1	1
	Fair	43	11	0.99 (0.71 to 1.39)	1.01 (071 to 1.44)
	Poor	17	16	0.46 (0.27 to 0.76)	0.41 (0.24 to 0.69)***

Significant at *p<0.05, **p<0.01, ***p<0.001.

## Discussion

This study revealed that being underweight at baseline and poor adherence to treatment had a significant negative association with sputum culture conversion. Furthermore, resistance to two or more anti-TB medications and receiving a short DR-TB regimen are positively associated with sputum culture conversion.

In this study, 80% of DR-TB patients achieved their sputum culture conversion in their course of treatment. The median sputum conversion time was 3 months (IQR 2–7). This finding is comparable with studies conducted in South Korea,^26^ China^27^ and a meta-analysis of studies conducted in seven countries,^17^ reporting a median sputum conversion time of 3 months. The median time of sputum conversion in the present study is longer than that in the study conducted in eastern Ethiopia^28^ (2 months) and shorter than the that in study conducted in Nigeria^29^ (5.5 months) and in the USA^30^ (6 months). Inconsistent findings with some previous studies might be due to early or delayed initiation of treatment, variations in sample size and participant characteristics and different definition criteria for the outcome of interest. In addition, using different measurements for outcome variables may also be a reason for varying results.

The present study revealed that DR-TB patients who were underweight (BMI <18.5 kg/m^2^) at baseline had late/low sputum culture conversion rate (aHR 0.65 [95% CI 0.49 to 0.90], p=0.01) compared with patients with a BMI ≥18.5 kg/m^2^. This finding is in line with the study conducted in Ethiopia^31^ (aHR 1.257) for delayed sputum culture conversion, in eastern Africa^32^ (aHR 0.32) and in Indonesia^33^ (aHR 0.55). Low BMI (<18.5 kg/m^2^) is an indicator of poor nutritional status or undernutrition. Malnutrition is known to alter the body’s immune function, and the accompanying reduced immunity increases susceptibility to various infectious diseases.^26,34^ Insufficient protein and caloric intake can impair the function of host defence mechanisms that are essential for recovering from TB disease. Therefore, malnutrition may increase the severity of infections and decrease medication effectiveness due to malabsorption and impaired metabolism. These may increase the duration of sputum culture conversion and the overall recovery from the disease. Furthermore, malnutrition increases the risk of adverse effects like hepatotoxicity, which may be related to poor adherence to TB treatment, and low BMI associated with a high risk of TB and HIV-related morbidity and mortality.^12,35^

This study revealed that patients who poorly adhered to TB treatment had a longer time to sputum conversion (aHR 0.41 [95% CI 0.24 to 0.69], p<0.01) compared with patients who had good adherence to TB treatment. This result agrees with the studies conducted in Nigeria^29^ and Rwanda^36^ showing treatment adherence has a relationship with sputum culture conversion. This may be because patients do not follow the required course of treatment, thus patients remain infectious for longer and are less likely to be cured of the disease. Furthermore, it may be due to a lack of adherence to interventions, and treatment supervision alone in the form of directly observed therapy is not effective in improving the time to sputum culture conversion and TB treatment outcomes.

In this study, the early sputum culture conversion was observed among patients resistant to two or more drugs (aHR 1.58 [95% CI 1.07 to 2.32], p=0.02) compared with patients resistant to a single drug (rifampicin). This finding is supported by a study conducted in Pakistan that reported TB patients resistant to two to four medications at treatment initiation had an early sputum culture conversion compared with patients resistant to a single drug.^18^ Similarly, the previous study conducted in Ethiopia showed that patients resistant to two or three drugs had a favourable outcome (cured and completed) compared with those resistant to a single drug.^37^ This may strengthen the present finding, as early sputum culture conversion is a primary predictor of overall end-treatment outcome.^12,18,38^ Even though a scientific reason for these findings is not clear, there may be possible reasons for this finding. First, it may be due to greater effectiveness of MDR-TB drugs in the treatment regimen, which is likely to improve treatment response in MDR-TB patients. In particular, fluoroquinolones have a significant role in curing MDR-TB patients and are used as a significant predictor of early culture conversion and end-treatment outcome.^12^ The second reason could be that in the present study, there was an earlier initiation of treatment based on professional experience and the patients’ history of previous TB treatment after rule outing resistance to rifampicin by using the Xpert MTB/RIF Assay without waiting for DST confirmation for second-line drugs. The third reason might be close follow-up by healthcare providers and the patients’ good compliance.

In addition, this study also revealed that DR-TB patients receiving shorter DR-TB regimens had a 24% higher probability of early sputum culture conversion (aHR 2.24 [95% CI 1.10 to 2.55], p=0.03) compared with patients on standardized longer treatment regimens. This finding is comparable with a study conducted in Kyrgyzstan (Central Asia) that revealed DR-TB patients on a short regimen had a 35% greater chance of sputum culture conversion with favourable outcomes in 83%.^39^ Another study reported that patients who met the criteria for the shorter DR-TB regimen had a higher likelihood of early sputum culture conversion and treatment success than patients receiving longer regimens.^28,40^ This may be due to the potential effectiveness of the shorter DR-TB treatment regimen (4 kanamycin–moxifloxacin–prothionamide–clofazimine–pyrazinamide–high-dose isoniazid–ethambutol/5 moxifloxacin–clofazimine–pyrazinamide–ethambutol). In particular, high-dose isoniazid may play a role in acting as a rapid bactericide. Based on WHO and national DR-TB treatment guidelines, effective anti-TB drugs are recommended for DR-TB patients for 9–12 months, excluding only confirmed resistance to second-line injectable medicines or fluoroquinolones and drugs included in the regimen. It was found that resistance to pyrazinamide or fluoroquinolones is an independent predictor for a longer sputum conversion.^41^ Furthermore, the difference in DR-TB treatment regimen composition influences the time to culture conversion.^35,36^

### Strengths and limitations of the study

The strengths of this study were using data from two different settings and using all available patients’ medical information for 9 y. However, the study has certain limitations, as some patient medical records were not included in the analysis because of inconsistent and incomplete information. In addition, the late introduction of a shorter treatment regimen and its eligibility criteria may affect the findings of the study.

## Conclusions

The overall median time of sputum culture conversion in this study was 3 months, which is shorter than the median 4 months recommended by the WHO. BMI <18.5 kg/m^2^, poor adherence to treatment, resistance to two or more TB drugs and a short DR-TB treatment regimen were found to be a predictors of time to sputum culture conversion. Strengthening and implementing nutrition assessment and all psychosocial support packages during directly observed treatment strategies may improve the delay of sputum conversion among underweight patients and those with poor treatment adherence. In addition, encouraging the use of short treatment regimens may increase the probability of sputum culture conversion and good treatment outcomes among DR-TB patients.

## Data Availability

All data used in this study are available from the corresponding author upon reasonable request.
